# Gastrointestinal dysfunction score for mortality prediction in intensive care unit patients with pre-existing digestive system disease: a prospective observational study

**DOI:** 10.3389/fnut.2026.1831897

**Published:** 2026-05-28

**Authors:** Yiming Shi, Shangling Zhu, Zhenghua Chen, Kang Chao, Zicheng Huang, Xiang Gao

**Affiliations:** 1 Department of Intensive Care Unit, The Sixth Affiliated Hospital, Sun Yat-sen University, Guangzhou, Guangdong, China; 2 Department of Rheumatology, The Sixth Affiliated Hospital, Sun Yat-sen University, Guangzhou, China; 3 Department of Colorectal Surgery, Department of General Surgery, The Sixth Affiliated Hospital, Sun Yat-sen University, Guangzhou, Guangdong, China; 4 Department of Gastroenterology, The Sixth Affiliated Hospital, Sun Yat-sen University, Guangzhou, Guangdong, China; 5 Guangdong Provincial Clinical Research Center for Digestive Diseases, Guangzhou, Guangdong, China

**Keywords:** acute gastrointestinal injury, critical illness, gastrointestinal dysfunction, GIDS, SOFA

## Abstract

**Background:**

Gastrointestinal (GI) dysfunction has been increasingly recognized as a common and clinically important problem in patients in the Intensive Care Unit (ICU). GI dysfunction may affect nutrition, immune function, and microbiome homeostasis, which has been associated with adverse outcomes in ICU patients. However, conventional organ failure scores such as the Sequential Organ Failure Assessment (SOFA) score do not adequately account for GI dysfunction, particularly in patients with pre-existing digestive system disease.

**Objective:**

This study aimed to evaluate the performance of the Gastrointestinal Dysfunction Score (GIDS) in adult ICU patients with pre-existing digestive system disease and to examine whether integrating GIDS with SOFA improves discrimination for 28-day mortality compared to SOFA alone.

**Methods:**

This single-center, prospective observational study included adult patients admitted to the ICU, excluding pregnancy, ICU readmission, cessation of active treatment, loss to follow-up and death within 24 h of ICU admission. Patients were stratified into GI and non-GI cohorts based on the presence of pre-existing digestive system disease at ICU admission. GIDS, SOFA and acute gastrointestinal irnjury (AGI) gades were recorded daily for the first ICU week. We compared baseline clinical characteristics and 28-day mortality between cohorts and evaluated whether adding GIDS to SOFA improved discrimination.

**Results:**

A total of 486 patients were included, with 359 in the GI cohort. Using the score of the first day in ICU, GIDS (AUC = 0.701) outperformed AGI (AUC = 0.614). When combined with SOFA, GIDS+SOFA had the highest AUC of 0.764, compared with SOFA alone (AUC = 0.739). In the GI cohort, GIDS+SOFA also demonstrated superior discrimination (AUC = 0.754) compared to SOFA alone (AUC = 0.723).

**Conclusion:**

GIDS provides a meaningful stratification of 28-day mortality risk, especially in patients with pre-existing digestive system disease. Adding GIDS to SOFA modestly improved discrimination for 28-day mortality and warrants external validation. These findings support further validation and potential incorporation of GIDS into clinical practice.

## 1 Introduction

Gastrointestinal (GI) dysfunction is increasingly recognized as a core component of critical illness rather than a secondary complication (1–3). The gut contributes to nutrition, epithelial barrier integrity, immune signaling, and host–microbiome homeostasis, all of which may be disrupted during shock, sepsis, and multi-organ dysfunction (4). In contemporary Intensive Care Unit (ICU) practice, GI manifestations—such as feeding intolerance (FI) (2), vomiting, diarrhea, abdominal distension, GI bleeding, and absence of bowel sounds—are common and clinically important (5). To address this gap, the Gastrointestinal Dysfunction Score (GIDS) was developed using multicenter observational data. The score emphasizes routinely observable GI signs and symptoms and is intended as a practical bedside measure for daily care and research. International efforts are ongoing to prospectively validate GIDS against patient-centered outcomes, reflecting the field's need for a standardized, clinically useful GI dysfunction metric (2). A recent review of critical care estimates that GI dysmotility affects approximately 60% of critically ill patients, and intra-abdominal hypertension and its deleterious effects are present in at least one-third of ICU patients, underscoring how frequently the GI system is involved in ICU trajectories (6, 7). Nevertheless, evidence remains limited in clinically important subpopulations in whom baseline GI pathology may complicate symptom interpretation and risk estimation, particularly in adult ICU patients with pre-existing digestive system disease (8).

Unlike other organ systems with widely adopted bedside grading tools, the GI system lacks a standardized scoring system to quantify GI dysfunction in ICU patients. For example, cardiac function is routinely graded by the NYHA class and nervous system function is evaluated by the Glasgow Coma Scale. Although GI dysfunction represents a significant burden, its assessment in ICU patients has not received adequate attention. Several studies have attempted to standardize terminology and bedside grading. The Working Group on Abdominal Problems of the European Society of Intensive Care Medicine proposed definitions for acute GI injury with four severity grades. It introduced operational concepts for FI and other GI symptoms (9). However, subsequent research emphasizes that ambiguous and heterogeneous measurement details continue to limit comparability across studies and hinder the development of general frameworks (10). In parallel, established organ dysfunction scores used in ICU research and clinical care provide limited representation of the GI system. The Sequential Organ Failure Assessment (SOFA) score, originally published in 1996, was designed to quantify dysfunction across six main organ systems and has become a cornerstone of epidemiology, trial design, and outcome benchmarking in critical care (11). Notably, a recent international consensus concluded that additional organ systems, including the GI system, could not be incorporated into contemporary organ dysfunction scoring frameworks. The panel cited limitations in feasibility, data quality, and validity of candidate variables, highlighting a persistent measurement gap (12).

Adult ICU patients with pre-existing digestive system disease constitute a distinct high-risk subgroup in whom baseline GI pathology is associated with adverse ICU outcomes. In a large sepsis ICU cohort, pre-existing cirrhosis independently increased the odds of 30-day mortality, underscoring the prognostic weight of antecedent digestive disease beyond acute physiology alone (13). Similarly, critically ill patients with inflammatory bowel disease experience meaningful post-ICU mortality (16.3% within 90 days), often requiring organ support and parenteral nutrition (14). These data motivate dedicated validation of GI-focused scores in the population with pre-existing digestive system disease.

Accordingly, we evaluated the discrimination of GIDS for 28-day mortality in adult ICU patients with and without digestive system disease and assessed whether adding GIDS to SOFA improves discrimination compared with SOFA alone.

## 2 Method

### 2.1 Participants

This single-center, prospective observational study was conducted in the general ICU of The Sixth Affiliated Hospital of Sun Yat-sen University. Patients (aged ≥18 years) admitted between August 1, 2024, and January 29, 2025, were enrolled and followed for 28 days after ICU admission; the last follow-up was completed on February 27, 2025. Exclusion criteria were ICU readmission during the same hospitalization, cessation of active treatment, pregnancy, loss to follow-up, and death within 24 h of ICU admission. The protocol was approved by the Ethics Committee of The Sixth Affiliated Hospital of Sun Yat-sen University (2024ZSLYEC-363). Informed consent was waived due to the observational design and the use of routinely collected clinical data. All care was delivered in accordance with standard clinical practice, without study-mandated interventions. For prespecified subgroup analyses, patients were categorized according to documented pre-existing digestive system disease at ICU admission into the GI cohort and the non-GI cohort. Pre-existing digestive system diseases included GI bleeding, GI perforation, intra-abdominal infection, GI malignancy, intestinal obstruction, hepatobiliary calculi, liver cirrhosis, radiation enteritis, hernia, perianal abscess, pancreatitis, inflammatory bowel disease, and ischemic colitis.

### 2.2 Data collection

Two trained clinicians prospectively collected data using standardized case report forms to ensure data accuracy. Baseline variables recorded at ICU admission included age, sex, body mass index (BMI), primary reason for ICU admission, presence of pre-existing digestive system disease, APACHE II (15) score and SOFA score. The GIDS and AGI grades were recorded daily during the first week in ICU (ICU days 1–7). During hospitalization, the ICU stay time, hospital stay time, 28-day all-cause mortality and use of continuous renal replacement therapy (CRRT), vasoactive agents, mechanical ventilation were recorded.

### 2.3 GIDS assessment

Before study initiation, all investigators received standardized training in GIDS assessment; the definition is provided in Supplementary Table 1. During the first ICU week, GI and abdominal symptoms (including vomiting, regurgitation, diarrhea, abdominal distension, absent bowel sounds, GI bleeding, GI paralysis and intestinal obstruction), gastric residual volume, intra-abdominal pressure, nutritional status, and GI medications were recorded daily. Severe diarrhea was defined as Bristol Stool Scale types 6–7, with≥5 episodes per day or stool output ≥1,000 ml per day.

### 2.4 Nutrition protocol

For critically ill patients who were unable to maintain adequate oral intake within 24–48h after ICU admission and had a functional gastrointestinal tract, enteral nutrition (EN) was initiated according to current guideline recommendations (16, 17). EN was administered as continuous infusion via a nasogastric tube at an initial rate of 25 ml/h. FI was assessed every 6h after EN initiation. In the absence of FI, the infusion rate was increased by 20 ml/h until a target rate of 100 ml/h was reached. If FI occurred, prokinetic agents were administered and EN tolerance was reassessed 2h later; if intolerance persisted, the infusion rate was reduced by 10 ml/h and further adjusted after reassessment 6h later (16, 17). A standard polymeric formula was used as the initial EN formula. Peptide-based or high-protein formulas were selected in patients with malabsorption, persistent FI, or increased protein requirements, according to clinical assessment (16, 17). EN was reduced or interrupted in cases of vomiting or regurgitation, severe abdominal distension, increased aspiration risk, hemodynamic instability, procedure-related requirements, or markedly increased gastric residual/aspirate volume. A gastric residual/aspirate volume >500 ml/6h was used as the reference threshold for delayed or adjusted EN (16). Caloric targets were determined according to nutritional risk stratification: patients with a NUTRIC score ≥5 were prescribed 25–30 kcal/kg/day during the first week, whereas those with a NUTRIC score < 5 were prescribed 25 kcal/kg/day. The protein target was approximately 1.3 g/kg/day and was adjusted according to renal function, catabolic status, disease severity, and clinical tolerance (16). For patients at high nutritional risk, supplementary parenteral nutrition was provided if EN failed to meet the target energy and/or protein intake because of FI or other clinical reasons. Supplementary parenteral nutrition was not routinely used during the first week in patients at low nutritional risk (16, 17).

### 2.5 Statistical analysis

Sample size estimation was based on prior studies, assuming an area under the curve (AUC) of 0.750 with standard deviation (SD) about 0.25 for SOFA in predicting 28-day mortality. An absolute 8% increase in predictive performance was hypothesized after incorporating GI dysfunction scoring into SOFA. With α = 0.05 and β = 0.20, 213 patients were required; allowing for a 10% attrition rate, the target enrollment was at least 235 patients. Calculations were performed using R 4.4.3 and SPSS Statistics 25.0.

Categorical variables are presented as counts (percentages) and compared using the χ^2^ test or Fisher's exact test, as appropriate. Continuous variables are presented as mean (SD) if normally distributed and median (Interquartile Range) otherwise; comparisons between group used the *t*-test/analysis of variance, or the Mann–Whitney U or Kruskal–Wallis test, as appropriate. The GIDS, AGI, and SOFA scores used for both baseline analysis and multivariable regression models were based on data collected on the first day of admission. Univariable and multivariate analyses were performed with 28-day mortality as the outcome. To assess potential multicollinearity among variables included in the multivariable logistic regression models, collinearity diagnostics were performed using tolerance and variance inflation factor. Variables with variance inflation factor >5 or tolerance < 0.2 were considered to indicate problematic multicollinearity. Because AGI and GIDS both reflect gastrointestinal function, additional sensitivity analyses were performed by entering them separately into multivariable logistic regression models.

Discrimination for 28-day mortality was evaluated using receiver operating characteristic (12) curves for GIDS, AGI grade and SOFA, and for combined scores (AGI+SOFA and GIDS+SOFA); ROC significance was tested against the null hypothesis AUC = 0.5, and differences are reported descriptively. For clinical ease of use, the combined scores were developed using a simple additive scoring method. Clinical utility for predicting 28-day mortality was assessed using decision curve analysis (DCA) for GIDS, SOFA, and the combined score (GIDS+SOFA). All tests were 2-sided, and *P* < 0.05 was considered statistically significant.

## 3 Results

### 3.1 Baseline characteristics of clinically relevant information in cohorts

**Figure 1:**
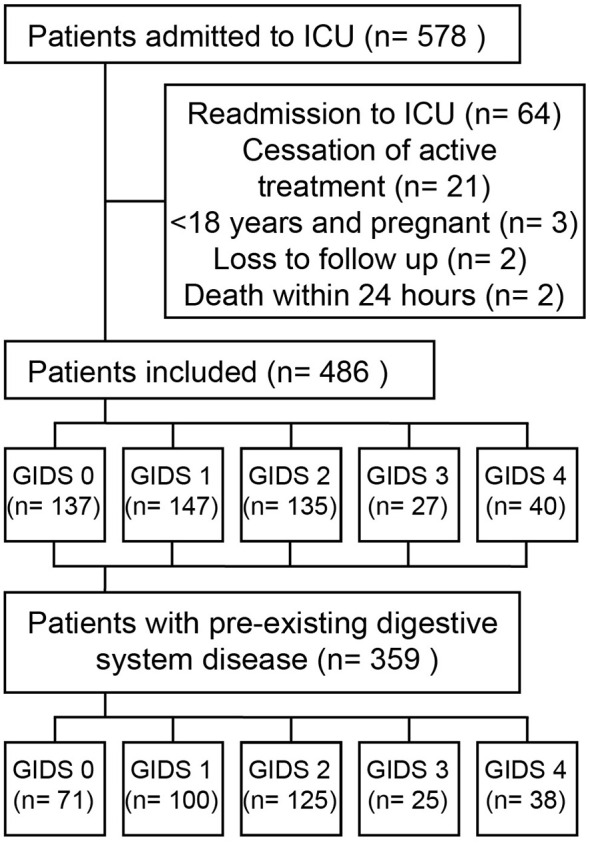
Study flowchart. Flow diagram of patient screening, exclusions, final inclusion (*n* = 486), and allocation into GIDS grades 0–4.

As shown in Figure 1, after exclusions for ICU readmission, cessation of active treatment, age < 18 years or pregnancy, loss to follow-up, and death within 24 h of ICU admission, 486 patients were finally included in our analysis. Patients were classified into GIDS grades 0–4 as follows: grade 0 (*n* = 137), grade 1 (*n* = 147), grade 2 (*n* = 135), grade 3 (*n* = 27), and grade 4 (*n* = 40).

As shown in Table 1, when stratified by pre-existing digestive system disease status, 359 patients comprised the GI cohort and 127 patients comprised the non-GI cohort. Significant differences were observed between cohorts: the GI cohort included a higher proportion of males (*P* = 0.003), a lower BMI (*P* < 0.001), and longer ICU stay time (*P*<*0*.001). The prevalence of stroke, sepsis, and vasoactive agents also differed significantly between cohorts (all *P* < 0.001). Distributions of SOFA, GIDS grades and AGI grades differed markedly between cohorts (all *P* < 0.005), with higher grades more frequently observed in the GI cohort. In contrast, 28-day mortality did not differ significantly between the GI and the non-GI cohorts (*P* = 0.152).

**Table 1:** Baseline characteristics of the overall cohort stratified by pre-existing digestive system disease status.

Variate	Total (*n* = 486)	Non-GI (*n* = 127)	GI (*n* = 359)	Statistic	*P*
15.6-7.2,-1.3498ptAge, M (IQR)	62.00 (51.00, 72.00)	59.00 (49.00, 70.00)	62.00 (52.00, 72.00)	Z = −1.51	0.131
**Gender**, ***n*** **(%)**					
Female	156 (32.10)	54 (42.52)	102 (28.41)	χ^2^ = 8.57	**0.003**
Male	330 (67.90)	73 (57.48)	257 (71.59)		
BMI, M (IQR)	21.26 (19.53, 24.15)	22.22 (20.61, 25.03)	20.81 (18.93, 23.85)	Z = −4.03	**< .001**
APACHEII, M (IQR)	19.00 (14.00, 25.00)	19.00 (14.00, 25.00)	19.00 (14.00, 26.00)	Z = −0.18	0.857
ICU stay time, M (IQR)	2.88 (1.55, 5.60)	2.00 (1.45, 5.59)	3.07 (1.71, 5.60)	Z = −3.30	**< .001**
Hospital stay time, M (IQR)	18.00 (11.00, 26.00)	17.00 (12.00, 24.50)	18.00 (10.00, 26.50)	Z = −0.46	0.649
15.6-7.2,-1.3498ptSOFA, M (IQR)	5.00 (0.00, 9.00)	4.00 (0.00, 7.50)	6.00 (0.00, 10.00)	Z = −2.93	**0.003**
**GIDS**, ***n*****(%)**					
Grade 0	137 (28.19)	66 (51.97)	71 (19.78)	χ^2^ = 75.76	**< .001**
Grade 1	147 (30.25)	47 (37.01)	100 (27.86)		
Grade 2	135 (27.78)	10 (7.87)	125 (34.82)		
Grade 3	27 (5.56)	2 (1.57)	25 (6.96)		
15.6-7.2,-1.3498ptGrade 4	40 (8.23)	2 (1.57)	38 (10.58)		
**AGI**, ***n*** **(%)**					
Grade 0	141 (29.01)	80 (62.99)	61 (16.99)	χ^2^ = 112.35	**< .001**
Grade 1	128 (26.34)	32 (25.20)	96 (26.74)		
Grade 2	117 (24.07)	13 (10.24)	104 (28.97)		
Grade 3	46 (9.47)	1 (0.79)	45 (12.53)		
15.6-7.2,-1.3498ptGrade 4	54 (11.11)	1 (0.79)	53 (14.76)		
**28-day mortality**, ***n*** **(%)**					
Survival	384 (79.01)	106 (83.46)	278 (77.44)	χ^2^ = 2.06	0.152
15.6-7.2,-1.3498ptDeath	102 (20.99)	21 (16.54)	81 (22.56)		
**Hypertension**, ***n*** **(%)**					
Without	339 (69.75)	81 (63.78)	258 (71.87)	χ^2^ = 2.91	0.088
15.6-7.2,-1.3498ptWith	147 (30.25)	46 (36.22)	101 (28.13)		
**Coronary Artery disease**, ***n*** **(%)**					
Without	423 (87.04)	108 (85.04)	315 (87.74)	χ^2^ = 0.61	0.435
15.6-7.2,-1.3498ptWith	63 (12.96)	19 (14.96)	44 (12.26)		
**Diabetes**, ***n*** **(%)**					
Without	402 (82.72)	99 (77.95)	303 (84.40)	χ^2^ = 2.73	0.099
15.6-7.2,-1.3498ptWith	84 (17.28)	28 (22.05)	56 (15.60)		
**Other Heart Disease**, ***n*** **(%)**					
Without	464 (95.47)	122 (96.06)	342 (95.26)	χ^2^ = 0.14	0.710
15.6-7.2,-1.3498ptWith	22 (4.53)	5 (3.94)	17 (4.74)		
**Pulmonary Disease**, ***n*** **(%)**					
Without	465 (95.68)	124 (97.64)	341 (94.99)	χ^2^ = 1.60	0.207
15.6-7.2,-1.3498ptWith	21 (4.32)	3 (2.36)	18 (5.01)		
**Stroke**, ***n*** **(%)**					
Without	453 (93.21)	110 (86.61)	343 (95.54)	χ^2^ = 11.82	**< .001**
15.6-7.2,-1.3498ptWith	33 (6.79)	17 (13.39)	16 (4.46)		
**Sepsis**, ***n*** **(%)**					
Without	301 (61.93)	99 (77.95)	202 (56.27)	χ^2^ = 18.71	**< .001**
15.6-7.2,-1.3498ptWith	185 (38.07)	28 (22.05)	157 (43.73)		
**Mechanical Ventilation**, ***n*** **(%)**					
Without	195 (40.12)	43 (33.86)	152 (42.34)	χ^2^ = 2.81	0.094
15.6-7.2,-1.3498ptWith	291 (59.88)	84 (66.14)	207 (57.66)		
**Vasoactive agents**, ***n*** **(%)**					
Without	262 (53.91)	89 (70.08)	173 (48.19)	χ^2^ = 18.09	**< .001**
15.6-7.2,-1.3498ptWith	224 (46.09)	38 (29.92)	186 (51.81)		
**CRRT**, ***n*** **(%)**					
Without	420 (86.42)	111 (87.40)	309 (86.07)	χ^2^ = 0.14	0.707
With	66 (13.58)	16 (12.60)	50 (13.93)		

Z, Mann-Whitney test; χ^2^, Chi-square test; M, median; IQR, interquartile range.

BMI, body mass index; SOFA, Sequential Organ Failure Assessment; GIDS, Gastrointestinal dysfunction score; AGI, acute gastrointestinal injury; CRRT, continuous renal replacement therapy.

**Figure 2:**
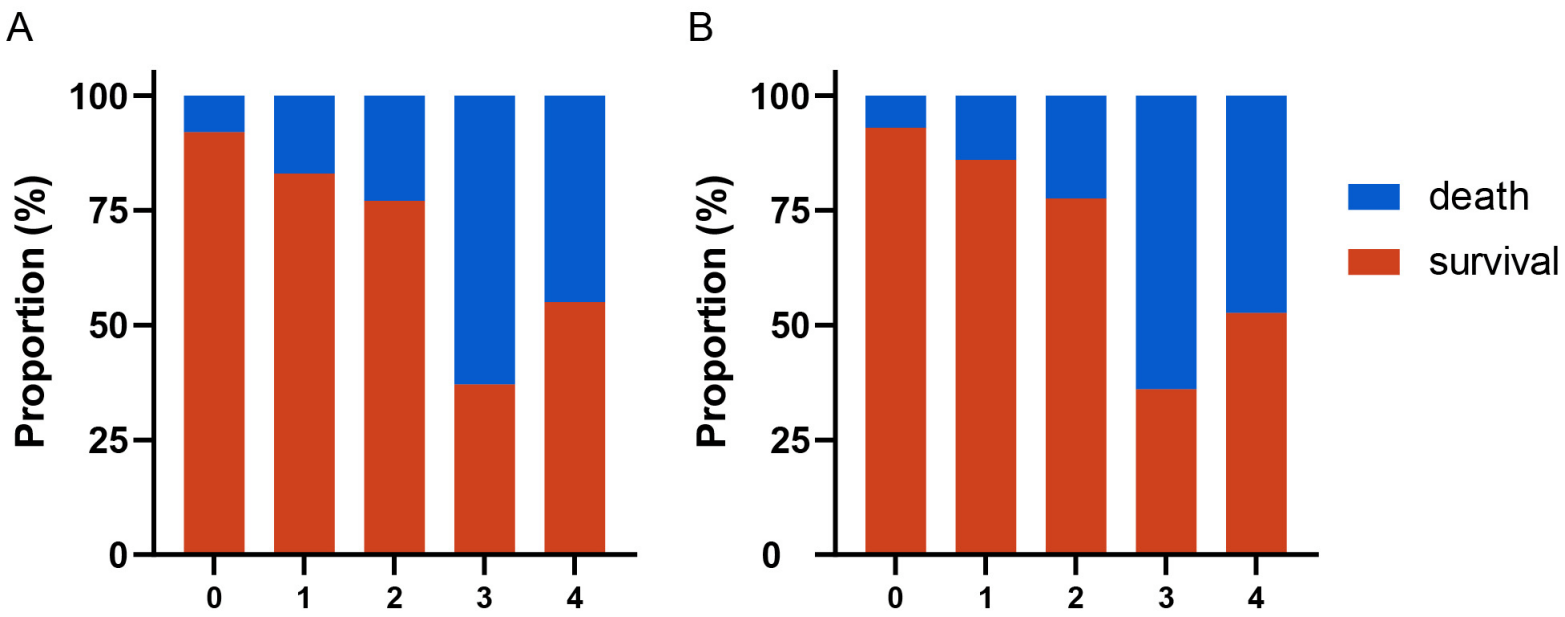
28-day mortality across GIDS grades. Bar plots show the distribution of 28-day mortality across GIDS grades 0–4 in **(A)** the overall cohort and **(B)** the GI cohort.

Baseline characteristics stratified by GIDS grade on the first day in ICU in the overall cohort are summarized in Table 2. Across increasing GIDS grades, APACHE II, AGI, SOFA and hospital stay time differed significantly (*P* < 0.001, *P* < 0.001, *P* < 0.001 and *P* = 0.025). Importantly, 28-day mortality also differed significantly across GIDS grades (*P* < 0.001), with higher mortality observed in grade 3 (Figure 2A). Mortality peaked at grade 3 potentially reflecting small sample sizes and case-mix differences in higher grades. In addition, diabetes (*P* = 0.009), sepsis (*P* < 0.001), mechanical ventilation (*P* = 0.010), vasoactive agents (*P* < 0.001), and CRRT (*P* = 0.046) differed across GIDS grades in the overall cohort.

**Table 2:** Baseline characteristics of the overall cohort stratified by GIDS grade.

Variate	Total (*n* = 486)	0 (*n* = 137)	1 (*n* = 147)	2 (*n* = 135)	3 (*n* = 27)	4 (*n* = 40)	Statistic	*P*
15.6-7.5,-1.3690ptAge, M (IQR)	62.00 (51.00, 72.00)	60.00 (49.00, 70.00)	64.00 (52.00, 71.00)	64.00 (50.50, 73.50)	61.00 (55.00, 69.50)	58.00 (48.75, 73.50)	χ^2^ = 2.55^#^	0.636
**Gender**, ***n*** **(%)**								
Female	156 (32.10)	44 (32.12)	42 (28.57)	47 (34.81)	8 (29.63)	15 (37.50)	χ^2^ = 1.91	0.753
Male	330 (67.90)	93 (67.88)	105 (71.43)	88 (65.19)	19 (70.37)	25 (62.50)		
BMI, M (IQR)	21.26 (19.53, 24.15)	21.56 (19.92, 24.50)	22.10 (20.02, 24.58)	20.76 (18.58, 23.97)	20.76 (18.93, 23.88)	20.76 (18.18, 22.01)	χ^2^ = 15.90^#^	**0.003**
APACHEII, M (IQR)	19.00 (14.00, 25.00)	18.00 (13.00, 24.00)	17.00 (13.00, 24.00)	20.00 (15.00, 26.00)	22.00 (18.00, 30.00)	22.00 (15.75, 29.50)	χ^2^ = 21.29^#^	**< .001**
ICU stay time, M (IQR)	2.88 (1.55, 5.60)	2.47 (1.48, 5.65)	2.71 (1.51, 4.66)	3.42 (1.69, 5.50)	3.45 (2.61, 7.76)	3.00 (2.22, 6.89)	χ^2^ = 8.67^#^	0.070
Hospital stay time, M (IQR)	18.00 (11.00, 26.00)	19.00 (13.00, 27.00)	18.00 (11.00, 26.00)	19.00 (11.00, 26.50)	14.00 (8.00, 20.00)	10.00 (7.00, 23.25)	χ^2^ = 11.13^#^	**0.025**
15.6-7.5,-1.3690ptSOFA, M (IQR)	5.00 (0.00, 9.00)	3.00 (0.00, 7.00)	5.00 (0.00, 9.00)	6.00 (1.00, 10.50)	10.00 (3.00, 15.50)	6.00 (2.00, 12.00)	χ^2^ = 23.25^#^	**< .001**
**Pre-existing digestive system disease**, ***n*** **(%)**								
Without	127 (26.13)	66 (48.18)	47 (31.97)	10 (7.41)	2 (7.41)	2 (5.00)	χ^2^ = 75.76	**< .001**
15.6-7.5,-1.3690ptWith	359 (73.87)	71 (51.82)	100 (68.03)	125 (92.59)	25 (92.59)	38 (95.00)		
**AGI**, ***n*** **(%)**								
Grade 0	141 (29.01)	104 (75.91)	30 (20.41)	5 (3.70)	1 (3.70)	1 (2.50)	χ^2^ = 472.22	**< .001**
Grade 1	128 (26.34)	24 (17.52)	75 (51.02)	26 (19.26)	2 (7.41)	1 (2.50)		
Grade 2	117 (24.07)	7 (5.11)	33 (22.45)	66 (48.89)	6 (22.22)	5 (12.50)		
Grade 3	46 (9.47)	1 (0.73)	4 (2.72)	26 (19.26)	10 (37.04)	5 (12.50)		
15.6-7.5,-1.3690ptGrade 4	54 (11.11)	1 (0.73)	5 (3.40)	12 (8.89)	8 (29.63)	28 (70.00)		
**28-day mortality**, ***n*** **(%)**								
Survival	384 (79.01)	126 (91.97)	122 (82.99)	104 (77.04)	10 (37.04)	22 (55.00)	χ^2^ = 58.19	**< .001**
15.6-7.5,-1.3690ptDeath	102 (20.99)	11 (8.03)	25 (17.01)	31 (22.96)	17 (62.96)	18 (45.00)		
**Hypertension**, ***n*** **(%)**								
15.6-7.5,-1.3690ptWithout	339 (69.75)	92 (67.15)	98 (66.67)	99 (73.33)	19 (70.37)	31 (77.50)	χ^2^ = 3.07	0.547
**Other Heart Disease**, ***n*** **(%)**								
Without	464 (95.47)	132 (96.35)	142 (96.60)	129 (95.56)	23 (85.19)	38 (95.00)	χ^2^ = 7.31	0.120
15.6-7.5,-1.3690ptWith	22 (4.53)	5 (3.65)	5 (3.40)	6 (4.44)	4 (14.81)	2 (5.00)		
**Pulmonary Disease**, ***n*** **(%)**								
Without	465 (95.68)	131 (95.62)	142 (96.60)	127 (94.07)	25 (92.59)	40 (100.00)	χ^2^ = 3.57	0.467
With	21 (4.32)	6 (4.38)	5 (3.40)	8 (5.93)	2 (7.41)	0 (0.00)		
**Stroke**, ***n*** **(%)**								
Without	453 (93.21)	122 (89.05)	140 (95.24)	127 (94.07)	25 (92.59)	39 (97.50)	χ^2^ = 6.04	0.196
15.6-7.5,-1.3690ptWith	33 (6.79)	15 (10.95)	7 (4.76)	8 (5.93)	2 (7.41)	1 (2.50)		
**Sepsis**, ***n*** **(%)**								
Without	301 (61.93)	97 (70.80)	105 (71.43)	61 (45.19)	11 (40.74)	27 (67.50)	χ^2^ = 31.92	**< .001**
15.6-7.5,-1.3690ptWith	185 (38.07)	40 (29.20)	42 (28.57)	74 (54.81)	16 (59.26)	13 (32.50)		
**Mechanical Ventilation**, ***n*** **(%)**								
Without	195 (40.12)	64 (46.72)	69 (46.94)	41 (30.37)	7 (25.93)	14 (35.00)	χ^2^ = 13.37	**0.010**
15.6-7.5,-1.3690ptWith	291 (59.88)	73 (53.28)	78 (53.06)	94 (69.63)	20 (74.07)	26 (65.00)		
**Vasoactive agents**, ***n*** **(%)**								
Without	262 (53.91)	89 (64.96)	86 (58.50)	61 (45.19)	9 (33.33)	17 (42.50)	χ^2^ = 18.82	**< .001**
15.6-7.5,-1.3690ptWith	224 (46.09)	48 (35.04)	61 (41.50)	74 (54.81)	18 (66.67)	23 (57.50)		
**CRRT**, ***n*** **(%)**								
Without	420 (86.42)	124 (90.51)	132 (89.80)	112 (82.96)	20 (74.07)	32 (80.00)	χ^2^ = 9.67	**0.046**
With	66 (13.58)	13 (9.49)	15 (10.20)	23 (17.04)	7 (25.93)	8 (20.00)		

#, Kruskal-Waills test; χ^2^, Chi-square test; M, median; IQR, interquartile range.

BMI, body mass index; SOFA, Sequential Organ Failure Assessment; GIDS, Gastrointestinal dysfunction score; AGI, acute gastrointestinal injury; CRRT, continuous renal replacement therapy.

Within the GI cohort, baseline characteristics stratified by GIDS grade are shown in Table 3. SOFA, APACHE II and AGI differed significantly across GIDS grades (all *P* < 0.001), and hospital stay time also varied across grades (*P* = 0.033). Consistent with the overall cohort, 28-day mortality also differed significantly across GIDS grades (*P* < 0.001), with higher mortality observed in grade 3 (Figure 2B). In addition, diabetes (*P* = 0.019), sepsis (*P* < 0.001), mechanical ventilation (*P* < 0.001), and CRRT (*P* = 0.005) differed significantly across GIDS grades in the GI cohort.

**Table 3:** Baseline characteristics of the GI cohort stratified by GIDS grade.

Variate	Total (*n* = 359)	0 (*n* = 71)	1 (*n* = 100)	2 (*n* = 125)	3 (*n* = 25)	4 (*n* = 38)	Statistic	*P*
Age, M (IQR)	62.00 (52.00, 72.00)	62.00 (50.00, 70.00)	64.50 (53.75, 73.00)	63.00 (50.00, 73.00)	61.00 (57.00, 71.00)	58.00 (49.75, 72.50)	χ^2^ = 2.51^#^	0.642
**Gender**, ***n*** **(%)**								
Female	102 (28.41)	14 (19.72)	24 (24.00)	43 (34.40)	8 (32.00)	13 (34.21)	χ^2^ = 6.59	0.159
Male	257 (71.59)	57 (80.28)	76 (76.00)	82 (65.60)	17 (68.00)	25 (65.79)		
BMI, M (IQR)	20.81 (18.93, 23.85)	20.76 (19.04, 23.85)	22.07 (19.52, 23.97)	20.76 (18.37, 24.00)	20.76 (19.92, 23.88)	20.76 (18.39, 21.90)	χ^2^ = 7.99^#^	0.092
APACHEII, M (IQR)	19.00 (14.00, 26.00)	17.00 (13.50, 23.00)	16.00 (12.00, 22.25)	20.00 (15.00, 26.00)	22.00 (18.00, 30.00)	22.50 (16.50, 30.50)	χ^2^ = 25.74^#^	**< .001**
ICU stay time, M (IQR)	3.07 (1.71, 5.60)	3.00 (1.65, 5.51)	3.00 (1.65, 4.66)	3.42 (1.70, 5.51)	3.53 (2.69, 7.83)	3.00 (2.12, 7.44)	χ^2^ = 5.19^#^	0.268
Hospital stay time, M (IQR)	18.00 (10.00, 26.50)	22.00 (13.00, 28.00)	18.00 (11.75, 28.25)	19.00 (11.00, 26.00)	14.00 (8.00, 20.00)	10.00 (7.00, 23.75)	χ^2^ = 10.45^#^	**0.033**
15.6-7.5,-1.3690ptSOFA, M (IQR)	6.00 (0.00, 10.00)	3.00 (0.00, 8.00)	5.00 (0.00, 9.00)	6.00 (1.00, 11.00)	11.00 (5.00, 16.00)	6.50 (2.25, 12.00)	χ^2^ = 19.12^#^	**< .001**
**AGI**, ***n*** **(%)**								
Grade 0	61 (16.99)	46 (64.79)	11 (11.00)	3 (2.40)	1 (4.00)	0 (0.00)	χ^2^ = 347.52	**< .001**
Grade 1	96 (26.74)	18 (25.35)	53 (53.00)	22 (17.60)	2 (8.00)	1 (2.63)		
Grade 2	104 (28.97)	5 (7.04)	28 (28.00)	63 (50.40)	4 (16.00)	4 (10.53)		
Grade 3	45 (12.53)	1 (1.41)	4 (4.00)	25 (20.00)	10 (40.00)	5 (13.16)		
15.6-7.5,-1.3690ptGrade 4	53 (14.76)	1 (1.41)	4 (4.00)	12 (9.60)	8 (32.00)	28 (73.68)		
**28–day mortality**, ***n*** **(%)**								
Survival	278 (77.44)	66 (92.96)	86 (86.00)	97 (77.60)	9 (36.00)	20 (52.63)	χ^2^ = 51.94	**< .001**
15.6-7.5,-1.3690ptDeath	81 (22.56)	5 (7.04)	14 (14.00)	28 (22.40)	16 (64.00)	18 (47.37)		
**Hypertension**, ***n*** **(%)**								
Without	258 (71.87)	51 (71.83)	70 (70.00)	91 (72.80)	17 (68.00)	29 (76.32)	χ^2^ = 0.78	0.941
15.6-7.5,-1.3690ptWith	101 (28.13)	20 (28.17)	30 (30.00)	34 (27.20)	8 (32.00)	9 (23.68)		
**Coronary Artery disease**, ***n*** **(%)**								
Without	315 (87.74)	67 (94.37)	82 (82.00)	108 (86.40)	23 (92.00)	35 (92.11)	χ^2^ = 7.27	0.122
15.6-7.5,-1.3690ptWith	44 (12.26)	4 (5.63)	18 (18.00)	17 (13.60)	2 (8.00)	3 (7.89)		
**Diabetes**, ***n*** **(%)**								
Without	303 (84.40)	61 (85.92)	78 (78.00)	109 (87.20)	18 (72.00)	37 (97.37)	χ^2^ = 11.75	**0.019**
15.6-7.5,-1.3690ptWith	56 (15.60)	10 (14.08)	22 (22.00)	16 (12.80)	7 (28.00)	1 (2.63)		
**Other Heart Disease**, ***n*** **(%)**								
Without	342 (95.26)	70 (98.59)	95 (95.00)	119 (95.20)	21 (84.00)	37 (97.37)	^*^	0.094
With	17 (4.74)	1 (1.41)	5 (5.00)	6 (4.80)	4 (16.00)	1 (2.63)		
Without	341 (94.99)	67 (94.37)	96 (96.00)	117 (93.60)	23 (92.00)	38 (100.00)	^*^	0.485
15.6-7.5,-1.3690ptWith	18 (5.01)	4 (5.63)	4 (4.00)	8 (6.40)	2 (8.00)	0 (0.00)		
**Stroke**, ***n*** **(%)**								
Without	343 (95.54)	67 (94.37)	97 (97.00)	119 (95.20)	23 (92.00)	37 (97.37)	^*^	0.713
15.6-7.5,-1.3690ptWith	16 (4.46)	4 (5.63)	3 (3.00)	6 (4.80)	2 (8.00)	1 (2.63)		
**Sepsis**, ***n*** **(%)**								
Without	202 (56.27)	44 (61.97)	69 (69.00)	54 (43.20)	10 (40.00)	25 (65.79)	χ^2^ = 20.29	**< .001**
15.6-7.5,-1.3690ptWith	157 (43.73)	27 (38.03)	31 (31.00)	71 (56.80)	15 (60.00)	13 (34.21)		
**Mechanical Ventilation**, ***n*** **(%)**								
Without	152 (42.34)	41 (57.75)	54 (54.00)	39 (31.20)	6 (24.00)	12 (31.58)	χ^2^ = 24.07	**< .001**
15.6-7.5,-1.3690ptWith	207 (57.66)	30 (42.25)	46 (46.00)	86 (68.80)	19 (76.00)	26 (68.42)		
**Vasoactive**, ***n*** **(%)**								
Without	173 (48.19)	39 (54.93)	56 (56.00)	54 (43.20)	8 (32.00)	16 (42.11)	χ^2^ = 8.17	0.086
15.6-7.5,-1.3690ptWith	186 (51.81)	32 (45.07)	44 (44.00)	71 (56.80)	17 (68.00)	22 (57.89)		
**CRRT**, ***n*** **(%)**								
Without	309 (86.07)	67 (94.37)	92 (92.00)	102 (81.60)	18 (72.00)	30 (78.95)	χ^2^ = 14.83	**0.005**
With	50 (13.93)	4 (5.63)	8 (8.00)	23 (18.40)	7 (28.00)	8 (21.05)		

#, Kruskal-Waills test; χ^2^, Chi-square test; ^*^, Fisher exact; M, median; IQR, interquartile range.

BMI, body mass index; SOFA, Sequential Organ Failure Assessment; GIDS, Gastrointestinal dysfunction score; AGI, acute gastrointestinal injury; CRRT, continuous renal replacement therapy.

### 3.2 Discrimination of Individual Scores for 28-Day Mortality

**Figure 3:**
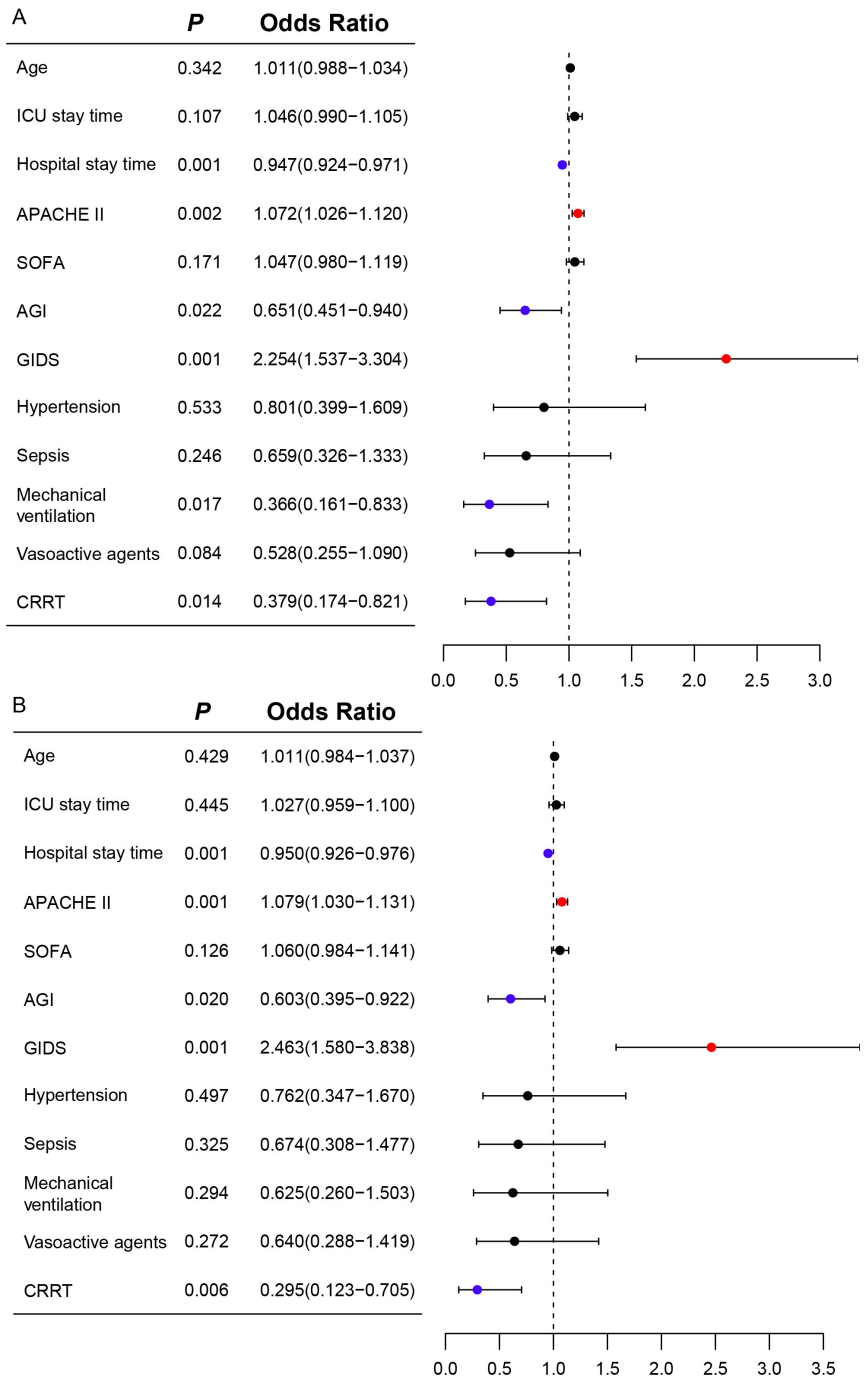
Multivariate logistic regression analysis for 28-day mortality. Forest plots show the odds ratio of 28-day mortality in **(A)** the overall cohort and **(B)** the GI cohort.

**Figure 4:**
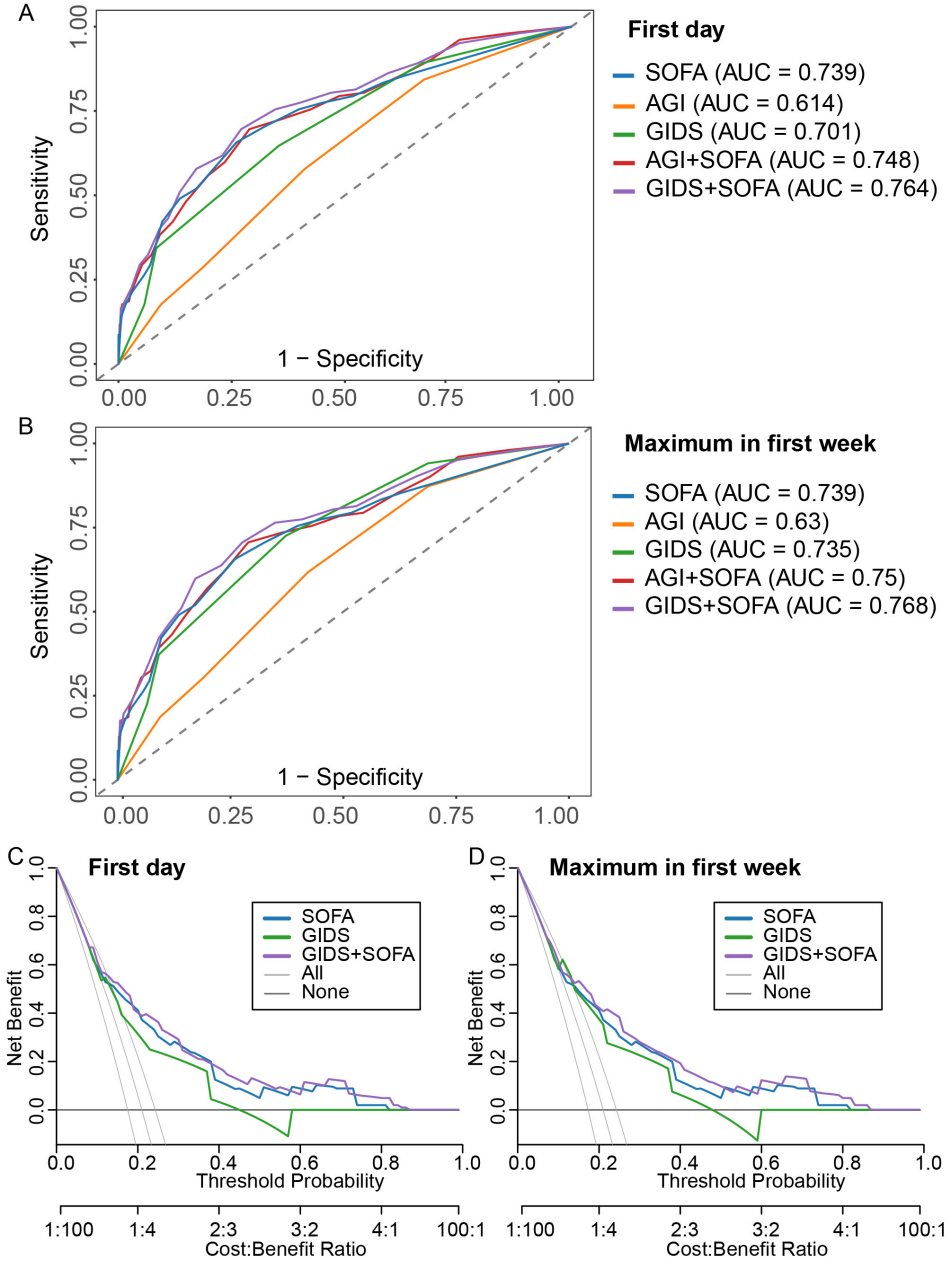
ROC curves and DCA curves for prediction of 28-day mortality in the overall cohort. **(A)** ROC curves comparing discrimination of first day in ICU SOFA, GIDS, AGI, AGI+SOFA, and GIDS+SOFA. **(B)** ROC curves comparing discrimination of maximum scores within the first ICU week of SOFA, GIDS, AGI, AGI+SOFA, and GIDS+SOFA. **(C)** DCA curves comparing clinical value of first day in ICU SOFA, GIDS, and GIDS+SOFA. **(D)** DCA curves comparing clinical value of maximum scores within the first ICU week of SOFA, GIDS, and GIDS+SOFA.

**Figure 5:**
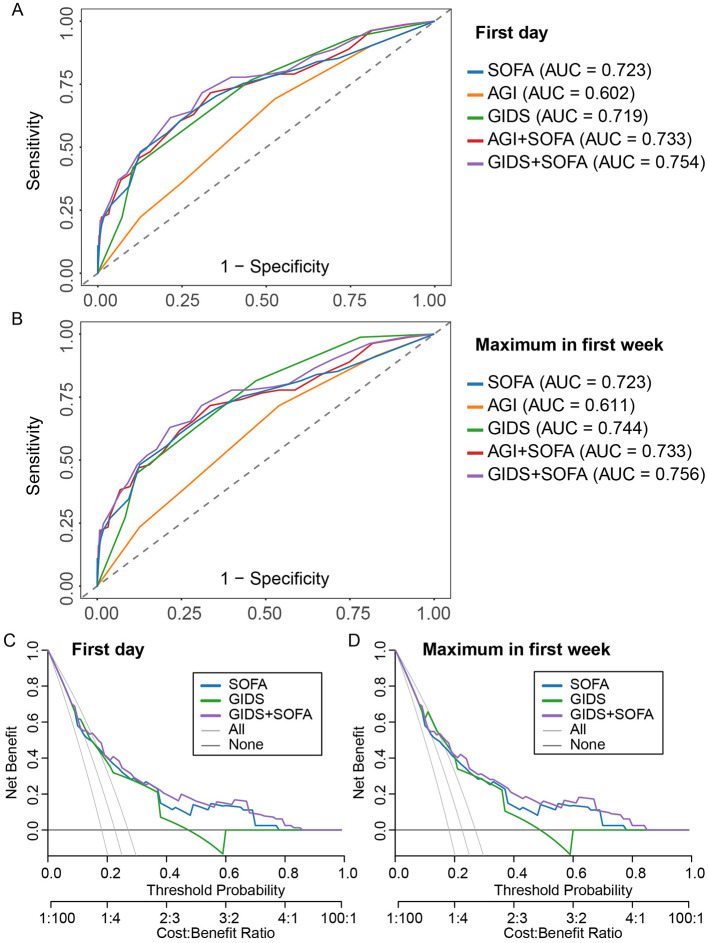
ROC curves and DCA curves for prediction of 28-day mortality in the GI cohort. **(A)** ROC curves comparing discrimination of first day in ICU SOFA, GIDS, AGI, AGI+SOFA, and GIDS+SOFA in the GI cohort. **(B)** ROC curves comparing discrimination of maximum scores within the first ICU week of SOFA, GIDS, AGI, AGI+SOFA, and GIDS+SOFA in the GI cohort. **(C)** DCA curves comparing clinical value of first day in ICU SOFA, GIDS, and GIDS+SOFA in the GI cohort. **(D)** DCA curves comparing clinical value of maximum scores within the first ICU week of SOFA, GIDS, and GIDS+SOFA in the GI cohort.

Univariate logistic regression analyses based on the data collected on the first day in ICU are presented in Supplementary Table 2. In the overall cohort, SOFA, AGI, and GIDS were significantly associated with 28-day mortality (all *P* ≤ 0.001). Similar associations were observed in the GI cohort (all *P* ≤ *0*.005). In multivariate logistic regression after controlling for confounding factors, GIDS remained independently associated with 28-day mortality in both cohorts (Figure 3A: the overall cohort: OR = 2.254, *P* = 0.001; Figure 3B: the GI cohort: OR = 2.463, *P* = 0.001). The collinearity diagnostics for multivariable logistic regression models are presented in Supplementary Table 3, which showed no evidence of severe multicollinearity among the covariates. In particular, AGI and GIDS had VIF values of 2.318 and 2.322 in the overall cohort, with nearly values of 2.209 and 2.256 were observed in the GI cohort, indicating an acceptable level of correlation. Separate multivariable models including AGI or GIDS individually for yielded similar results (Supplementary Table 4). ROC analyses comparing discrimination for 28-day mortality are shown in Figure 4 and Figure 5, with detailed numerical estimates provided in Supplementary Table 5. In the overall cohort on the first day in ICU, GIDS (AUC = 0.701) outperformed AGI (AUC = 0.614), and the GIDS+SOFA model achieved the highest AUC (AUC = 0.764), exceeding SOFA alone (AUC = 0.739) (Figure 4A). Using the maximum scores within the first ICU week, GIDS+SOFA remained the best-performing model (AUC = 0.768) (Figure 4B). In the GI cohort, a similar pattern was observed. On first day in ICU, GIDS+SOFA again demonstrated the highest discrimination (AUC = 0.754) comparing with other models (Figure 5A). Using the maximum scores within the first ICU week, GIDS+SOFA remained highest (AUC = 0.756) (Figure 5B). In conclusion, GIDS+SOFA consistently showed the strongest discrimination, suggesting that the combined use of GIDS and SOFA scores offers greater discrimination for identifying high-risk individuals in both the general population and patients with pre-existing digestive system disease.

### 3.3 Incremental clinical utility of GIDS+SOFA scores

Decision curve analyses (DCA) are shown in Figures 4C–D for the overall cohort and Figures 5C, D for the GI cohort. On first day in ICU, the GIDS+SOFA model provided a greater net benefit than SOFA alone or GIDS alone across a clinically relevant range of threshold probabilities (Figures 4C, 5C). Similar findings were observed when using the maximum scores within the first ICU week, with GIDS+SOFA demonstrating the most favorable net benefit compared with alternative models in both cohorts (Figures 4D, 5D). Across both cohorts, adding GIDS to SOFA increased AUC by approximately 0.02–0.04 and yielded higher net benefit than either score alone in decision-curve analyses.

## 4 Discussion

This prospective observational ICU study demonstrated a graded association between GIDS category and 28-day mortality. In both the overall cohort and the subgroup with pre-existing digestive system disease, models incorporating GIDS and SOFA showed numerically higher discrimination than SOFA alone (in the overall cohort, first day in ICU GIDS+SOFA (AUC = 0.764) vs. SOFA (AUC = 0.739); in the GI cohort, first day in ICU GIDS+SOFA (AUC = 0.754) vs. SOFA (AUC = 0.723)). In contrast, AGI alone consistently yielded the lowest performance across time windows. Similar patterns were observed when using the maximum value within the first ICU week, with GIDS+SOFA achieving the highest AUC in both cohorts. The AUC improvement with GIDS+SOFA was modest, suggesting that structured GI assessment may add complementary prognostic information.

GI dysfunction has long been recognized as a frequent and clinically relevant problem in critically ill patients (18, 19). According to a prospective multicenter study, the occurrence of GI symptoms during the first ICU week was independently associated with adverse outcomes (20). Despite this, contemporary organ dysfunction scores such as SOFA primarily capture systemic physiological derangements and do not explicitly incorporate GI manifestations commonly used in bedside decision-making, including FI, abdominal distension, GI bleeding, or abnormal intra-abdominal pressure. This selective omission may lead to the underestimation of GI functional burden in global severity assessment, highlighting a gap in routinely capturing GI dysfunction within widely used organ failure scores.

The GIDS, introduced in 2021, was developed to provide a structured, standardized quantification of GI signs and symptoms that can be readily obtained at the bedside (2). By translating heterogeneous clinical observations into graded categories, GIDS attempts to operationalize GI dysfunction in a standardized way that can be incorporated into prognostic models. In our study, the consistent numerical improvement observed when GIDS was added to SOFA supports the concept that GI dysfunction represents a dimension of organ failure not fully captured by traditional systemic scores.

The prognostic performance of GIDS has varied across previous research. A prospective study suggested that GIDS may reflect disease severity by extending the ICU stay time and improving 28-day mortality prediction when combined with SOFA (21), whereas a retrospective analysis reported that the maximum GIDS during the first week in ICU was predictive of 28-day mortality, but the GIDS score within 24 h lacked reliability (22). Several factors may account for these differences. First, GIDS includes variables dependent on clinical documentation and bedside assessment. In retrospective settings, early time-point evaluations may be particularly vulnerable to incomplete recording or variability in documentation practices. Second, because GIDS reflects a dynamic state of GI function decompensation rather than structural injury, its prognostic utility may depend on the temporal pattern of dysfunction within a given cohort. In populations where GI deterioration develops a delay during the ICU course, the first-week maximum GIDS may be more valuable than early measurements within 24 h. In contrast, in our cohort, both the first day in ICU GIDS and the first-week maximum were associated with 28-day mortality, suggesting that GI dysfunction occurred early and in close parallel with overall disease severity.

AGI is commonly used in the evaluation of GI dysfunction in current research (23–25). Comparison with the AGI grading system further contextualizes these findings. Proposed in 2012, AGI was designed to characterize GI dysfunction as part of multiple organ dysfunction syndrome (9). While conceptually important, AGI grading incorporates global clinical signs and symptoms and may be influenced by overall patient condition. In addition, its reliance on relatively broad descriptors may limit its reproducibility in research settings (24). In our study, AGI grade was associated with 28-day mortality, confirming its clinical relevance. However, the discriminatory performance of models including AGI was inferior to the performance of models including GIDS. GIDS employs a more structured, symptom-based framework, which may therefore reduce subjectivity while facilitating integration into existing evaluation systems. From a practical perspective, it does not appear more complex than AGI and may be feasible for routine ICU use following standardized training.

An important aspect of our analysis was the evaluation of GIDS within patients with pre-existing digestive system disease. Prior studies have suggested that incorporating GI variables into multi-organ assessment may improve risk stratification (26). However, whether GIDS truly captures organ-specific functional burden rather than merely reflecting the presence of pre-existing digestive system disease remains uncertain. The GI cohort in our study had a higher baseline prevalence of GI symptoms. If GIDS reflected underlying disease labels, its discriminatory capacity would be expected to diminish in this subgroup. After adjusting these factors, GIDS remained independently associated with 28-day mortality and provided incremental information beyond SOFA. These findings support the interpretation that GIDS more likely reflects the severity of acute GI decompensation rather than the existence of pre-existing digestive system disease. Our findings contribute to ongoing efforts to better define and quantify GI dysfunction in critical illness. Nevertheless, current evidence primarily supports the use of GIDS in critically ill or decompensated settings, and its applicability across other stages of GI disease remains to be evaluated. External validation in multicenter cohorts, evaluation of interrater reliability, and assessment of calibration and clinical utility are warranted before broader implementation.

Several limitations should be considered. First, GIDS relies on bedside assessment and may be susceptible to inter-observer variability; interrater reliability was not formally evaluated. Second, this single-center design may limit generalizability to ICUs with different case-mix and practice patterns. Third, relatively few patients were classified as GIDS grades 3–4, which may increase uncertainty in grade-specific estimates. Finally, residual confounding is possible despite multivariable modeling. Therefore, incremental discrimination should be interpreted descriptively, and future studies should evaluate calibration and clinical utility.

In summary, GIDS represents a structured approach to GI function assessment that may complement SOFA in predicting short-term outcomes in critically ill patients. Its performance within patients with pre-existing digestive system disease further suggests that it captures acute functional decompensation rather than underlying diagnostic categories alone. Continued investigation is required to determine its role within future multi-organ assessment frameworks.

## 5 Conclusion

In this prospective ICU cohort, higher GIDS grades were associated with higher 28-day mortality. Across both the overall cohort and the subgroup with pre-existing digestive system disease, ROC analyses showed that incorporating GIDS into SOFA yielded numerically higher AUCs than SOFA alone, whereas AGI alone demonstrated the weakest discrimination. These findings suggest that symptom-oriented quantification of GI dysfunction may complement conventional organ failure assessment and warrant multicenter validation, with attention to reproducibility and clinical utility, especially in patients with pre-existing digestive system disease.

## Data Availability

The raw data supporting the conclusions of this article will be made available by the authors, without undue reservation.
