# Supplementary material for: Gastrointestinal dysfunction score for mortality prediction in intensive care unit patients with pre-existing digestive system disease: a prospective observational study
Source: Front Nutr. 2026 May 28;13:1831897. doi: 10.3389/fnut.2026.1831897 (PMC13253419; doi:10.3389/fnut.2026.1831897)
Supplement: Supplementary file 1 [file Table_1.docx]

**Supplemental Table 1. Definitions of Gastrointestinal Dysfunction Score.**

| **0—No risk** | **1—Increased risk** | **2—GI dysfunction** | **3— GI failure** | **4—Life threatening** |
| --- | --- | --- | --- | --- |
| Less than one of the following: | Two of the following: | Three or more symptoms of score 1 or up to two of the following: | Three or more of the following: | One of the following: |
| Absent bowel sounds;  Vomiting;  Gastric residual volume >200 mL;  GI paralysis/ dynamic ileus; Abdominal distension;  Diarrhea (not severe);  GI bleeding without transfusion;  Intra-abdominal pressure >20 mmHg | No oral intake;  Absent bowel sounds;  Vomiting;  Gastric residual volume >200 mL;  GI paralysis/ dynamic ileus;  Abdominal distension;  Diarrhea (not severe);  GI bleeding without transfusion;  Intra-abdominal pressure >20 mmHg | Severe diarrhea;  GI bleeding with transfusion;  Intra-abdominal pressure >20 mmHg | Prokinetic use;  GI paralysis/ dynamic ileus;  Abdominal distension;  Severe diarrhea;  GI bleeding with transfusion;  Intra-abdominal pressure >20 mmHg | GI bleeding leading to hemorrhagic shock;  Mesenteric ischemia; Abdominal compartment syndrome |

GI, gastrointestinal.
