# Supplementary material for: Gastrointestinal dysfunction score for mortality prediction in intensive care unit patients with pre-existing digestive system disease: a prospective observational study
Source: Front Nutr. 2026 May 28;13:1831897. doi: 10.3389/fnut.2026.1831897 (PMC13253419; doi:10.3389/fnut.2026.1831897)
Supplement: Supplementary file 2 [file Table_2.docx]

**Supplemental Table 2. Univariate** **logistic regression analysis** **for 28-day mortality.**

| **Variable** | **Odds Ratio** | **Lower 95% CI** | **Higher 95% CI** | ***P*** |
| --- | --- | --- | --- | --- |
| **Overall Cohort** | | | | |
| Digestive system Disease | 0.68 | 0.4 | 1.155 | 0.153 |
| SOFA | 1.195 | 1.142 | 1.251 | **0.001** |
| AGI | 1.346 | 1.141 | 1.587 | **0.001** |
| GIDS | 1.855 | 1.535 | 2.241 | **0.001** |
| Gender | 1.074 | 0.675 | 1.708 | 0.764 |
| Age | 1.022 | 1.007 | 1.037 | **0.003** |
| BMI | 0.988 | 0.939 | 1.04 | 0.643 |
| Hypertension | 0.632 | 0.400 | 0.998 | **0.049** |
| Coronary Artery disease | 1.025 | 0.533 | 1.97 | 0.941 |
| Diabetes | 0.820 | 0.47 | 1.432 | 0.485 |
| Other Heart Disease | 0.552 | 0.219 | 1.391 | 0.208 |
| Pulmonary Disease | 0.650 | 0.246 | 1.721 | 0.386 |
| Stroke | 0.689 | 0.31 | 1.532 | 0.361 |
| Sepsis | 0.288 | 0.183 | 0.454 | **0.001** |
| APACHE II | 1.143 | 1.107 | 1.180 | **0.001** |
| ICU stay time | 1.097 | 1.058 | 1.136 | **0.001** |
| Hospital stay time | 0.970 | 0.953 | 0.988 | **0.001** |
| Mechanical Ventilation | 0.162 | 0.088 | 0.300 | **0.001** |
| Vasoactive agents | 0.143 | 0.084 | 0.243 | **0.001** |
| CRRT | 0.144 | 0.083 | 0.250 | **0.001** |
| **GI Cohort** | | | | |
| SOFA | 1.177 | 1.12 | 1.236 | **0.001** |
| AGI | 1.328 | 1.092 | 1.613 | **0.004** |
| GIDS | 1.996 | 1.592 | 2.504 | **0.001** |
| Gender | 1.353 | 0.795 | 2.305 | 0.265 |
| Age | 1.017 | 1.000 | 1,034 | 0.055 |
| BMI | 0.960 | 0.898 | 1.026 | 0.231 |
| Hypertension | 0.583 | 0.345 | 0.986 | **0.044** |
| Coronary Artery disease | 1.152 | 0.529 | 2.509 | 0.721 |
| Diabetes | 0.761 | 0.397 | 1.46 | 0.412 |
| Other Heart Disease | 0.394 | 0.145 | 1.072 | 0.068 |
| Pulmonary Disease | 1.021 | 0.327 | 3.191 | 0.972 |
| Stroke | 1.275 | 0.354 | 4.59 | 0.71 |
| Sepsis | 0.388 | 0.233 | 0.645 | **0.001** |
| APACHE II | 1.137 | 1.097 | 1.177 | **0.001** |
| ICU stay time | 1.084 | 1.037 | 1.133 | **0.001** |
| Hospital stay time | 0.969 | 0.949 | 0.989 | **0.002** |
| Mechanical Ventilation | 0.191 | 0.101 | 0.362 | **0.001** |
| Vasoactive agents | 0.208 | 0.116 | 0.373 | **0.001** |
| CRRT | 0.118 | 0.062 | 0.226 | **0.001** |

CI, confidence interval; BMI, body mass index; CRRT, continuous renal replacement therapy; AGI, acute gastrointestinal injury; GIDS, Gastrointestinal Dysfunction Score; SOFA, Sequential Organ Failure Assessment.
