# Supplementary material for: Gastrointestinal dysfunction score for mortality prediction in intensive care unit patients with pre-existing digestive system disease: a prospective observational study
Source: Front Nutr. 2026 May 28;13:1831897. doi: 10.3389/fnut.2026.1831897 (PMC13253419; doi:10.3389/fnut.2026.1831897)
Supplement: Supplementary file 3 [file Table_3.docx]

**Supplemental Table 3. Collinearity diagnostics for multivariable logistic regression models.**

| **Variable** | **Tolerance** | **Variance Inflation Factor** |
| --- | --- | --- |
| **Overall Cohort** | | |
| Age | 0.826 | 1.211 |
| ICU stay time | 0.648 | 1.542 |
| hospital stay time | 0.883 | 1.133 |
| APACHEII | 0.64 | 1.562 |
| SOFA | 0.655 | 1.527 |
| AGI | 0.431 | 2.318 |
| GIDS | 0.431 | 2.322 |
| Hypertension | 0.865 | 1.156 |
| Sepsis | 0.713 | 1.402 |
| Mechanical Ventilation | 0.696 | 1.437 |
| Vasoactive | 0.689 | 1.452 |
| CRRT | 0.808 | 1.237 |
| **GI Cohort** | | |
| Age | 0.853 | 1.173 |
| ICU stay time | 0.657 | 1.522 |
| hospital stay time | 0.863 | 1.159 |
| APACHEII | 0.649 | 1.54 |
| SOFA | 0.627 | 1.596 |
| AGI | 0.453 | 2.209 |
| GIDS | 0.443 | 2.256 |
| Hypertension | 0.907 | 1.102 |
| Sepsis | 0.777 | 1.287 |
| Mechanical Ventilation | 0.684 | 1.463 |
| Vasoactive | 0.735 | 1.361 |
| CRRT | 0.793 | 1.261 |

CRRT, continuous renal replacement therapy; AGI, acute gastrointestinal injury; GIDS, Gastrointestinal Dysfunction Score; SOFA, Sequential Organ Failure Assessment.
