# Supplementary material for: Gastrointestinal dysfunction score for mortality prediction in intensive care unit patients with pre-existing digestive system disease: a prospective observational study
Source: Front Nutr. 2026 May 28;13:1831897. doi: 10.3389/fnut.2026.1831897 (PMC13253419; doi:10.3389/fnut.2026.1831897)
Supplement: Supplementary file 4 [file Table_4.docx]

**Supplemental Table 4.** **Multivariable models for sensitivity analyses.**

| **Variable** | **Odds Ratio** | **Lower 95% CI** | **Higher 95% CI** | ***P*** |
| --- | --- | --- | --- | --- |
| **Overall Cohort** | | | | |
| **Multivariable model without GIDS** | | | | |
| Age | 1.007 | 0.986 | 1.029 | 0.512 |
| ICU stay time | 1.036 | 0.983 | 1.092 | 0.192 |
| hospital stay time | 0.949 | 0.926 | 0.972 | **0.001** |
| APACHEII | 1.082 | 1.038 | 1.129 | **0.001** |
| SOFA | 1.056 | 0.993 | 1.124 | 0.084 |
| AGI | 1.161 | 0.921 | 1.465 | 0.206 |
| Hypertension | 0.797 | 0.412 | 1.544 | 0.502 |
| Sepsis | 0.824 | 0.423 | 1.603 | 0.568 |
| Mechanical Ventilation | 0.391 | 0.179 | 0.853 | **0.018** |
| Vasoactive | 0.553 | 0.274 | 1.115 | 0.098 |
| CRRT | 0.351 | 0.166 | 0.745 | **0.006** |
| **Multivariable model without AGI** | | | | |
| Age | 1.008 | 0.986 | 1.031 | 0.469 |
| ICU stay time | 1.044 | 0.989 | 1.102 | 0.117 |
| hospital stay time | 0.949 | 0.926 | 0.972 | **0.001** |
| APACHEII | 1.076 | 1.031 | 1.123 | **0.001** |
| SOFA | 1.043 | 0.978 | 1.113 | 0.199 |
| GIDS | 1.618 | 1.259 | 2.079 | **0.001** |
| Hypertension | 0.731 | 0.370 | 1.447 | 0.369 |
| Sepsis | 0.753 | 0.380 | 1.493 | 0.417 |
| Mechanical Ventilation | 0.398 | 0.179 | 0.883 | **0.023** |
| Vasoactive | 0.529 | 0.258 | 1.085 | 0.082 |
| CRRT | 0.370 | 0.172 | 0.798 | **0.011** |
| **GI Cohort** | | | | |
| **Multivariable model without GIDS** | | | | |
| Age | 1.006 | 0.982 | 1.030 | 0.648 |
| ICU stay time | 1.023 | 0.958 | 1.092 | 0.504 |
| hospital stay time | 0.953 | 0.929 | 0.978 | **0.001** |
| APACHEII | 1.092 | 1.044 | 1.142 | **0.001** |
| SOFA | 1.063 | 0.991 | 1.139 | 0.087 |
| AGI | 1.136 | 0.874 | 1.476 | 0.341 |
| Hypertension | 0.684 | 0.330 | 1.419 | 0.308 |
| Sepsis | 0.948 | 0.459 | 1.958 | 0.886 |
| Mechanical Ventilation | 0.636 | 0.278 | 1.455 | 0.284 |
| Vasoactive | 0.657 | 0.305 | 1.415 | 0.283 |
| CRRT | 0.256 | 0.110 | 0.594 | **0.002** |
| **Multivariable model without AGI** | | | | |
| Age | 1.007 | 0.982 | 1.033 | 0.569 |
| ICU stay time | 1.018 | 0.953 | 1.088 | 0.592 |
| hospital stay time | 0.954 | 0.930 | 0.979 | **0.001** |
| APACHEII | 1.085 | 1.036 | 1.136 | **0.001** |
| SOFA | 1.058 | 0.984 | 1.138 | 0.128 |
| GIDS | 1.673 | 1.260 | 2.222 | **0.001** |
| Hypertension | 0.663 | 0.310 | 1.415 | 0.288 |
| Sepsis | 0.778 | 0.363 | 1.666 | 0.518 |
| Mechanical Ventilation | 0.692 | 0.296 | 1.613 | 0.393 |
| Vasoactive | 0.628 | 0.286 | 1.378 | 0.246 |
| CRRT | 0.285 | 0.121 | 0.671 | **0.004** |

CRRT, continuous renal replacement therapy; AGI, acute gastrointestinal injury; GIDS, Gastrointestinal Dysfunction Score; SOFA, Sequential Organ Failure Assessment.
