# Supplementary material for: Gastrointestinal dysfunction score for mortality prediction in intensive care unit patients with pre-existing digestive system disease: a prospective observational study
Source: Front Nutr. 2026 May 28;13:1831897. doi: 10.3389/fnut.2026.1831897 (PMC13253419; doi:10.3389/fnut.2026.1831897)
Supplement: Supplementary file 5 [file Table_5.docx]

**Supplemental Table 5.** **Predictive value for ROC curve of SOFA, AGI，GIDS, AGI+SOFA and GIDS+SOFA for 28-day mortality.**

| **Variable** | ***P*-value** | **AUC** | **Lower 95% CI** | **Higher 95% CI** |
| --- | --- | --- | --- | --- |
| **First Day in the overall cohort** | | | | |
| SOFA | <0.001 | 0.739 | 0.681 | 0.797 |
| AGI | 0.009 | 0.614 | 0.556 | 0.672 |
| GIDS | <0.001 | 0.701 | 0.645 | 0.757 |
| AGI+SOFA | <0.001 | 0.748 | 0.693 | 0.803 |
| GIDS+SOFA | <0.001 | 0.764 | 0.710 | 0.818 |
| **First Week in the overall cohort** | | | | |
| SOFA | <0.001 | 0.739 | 0.681 | 0.797 |
| AGI | <0.001 | 0.630 | 0.573 | 0.687 |
| GIDS | <0.001 | 0.735 | 0.684 | 0.786 |
| AGI+SOFA | <0.001 | 0.750 | 0.695 | 0.805 |
| GIDS+SOFA | <0.001 | 0.768 | 0.714 | 0.822 |
| **First Day in the GI cohort** | | | | |
| SOFA | <0.001 | 0.723 | 0.654 | 0.792 |
| AGI | 0.017 | 0.602 | 0.535 | 0.670 |
| GIDS | <0.001 | 0.719 | 0.658 | 0.780 |
| AGI+SOFA | <0.001 | 0.733 | 0.666 | 0.799 |
| GIDS+SOFA | <0.001 | 0.754 | 0.690 | 0.817 |
| **First Week in the GI cohort** | | | | |
| SOFA | <0.001 | 0.723 | 0.654 | 0.792 |
| AGI | 0.158 | 0.611 | 0.543 | 0.678 |
| GIDS | <0.001 | 0.744 | 0.689 | 0.799 |
| AGI+SOFA | <0.001 | 0.733 | 0.666 | 0.800 |
| GIDS+SOFA | <0.001 | 0.756 | 0.693 | 0.820 |

AUC, area under the curve; CI, confidence interval; AGI, acute gastrointestinal injury; GIDS, Gastrointestinal Dysfunction Score; SOFA, Sequential Organ Failure Assessment.
